# New Insights on the Effects of Dietary Omega-3 Fatty Acids on Impaired Skin Healing in Diabetes and Chronic Venous Leg Ulcers

**DOI:** 10.3390/foods10102306

**Published:** 2021-09-28

**Authors:** Simona Serini, Gabriella Calviello

**Affiliations:** Department of Translational Medicine and Surgery, Section of General Pathology, School of Medicine, Fondazione Policlinico Universitario A. Gemelli IRCCS—Università Cattolica del Sacro Cuore, L.go F. Vito, 1-00168 Roma, Italy; simona.serini@unicatt.it

**Keywords:** diabetes, epigenetics, innate immune cells, microbiota, Omega-3 PUFA, venous leg ulcers, wound healing

## Abstract

Long-chain Omega-3 polyunsaturated fatty acids (Omega-3 PUFAs) are widely recognized as powerful negative regulators of acute inflammation. However, the precise role exerted by these dietary compounds during the healing process is still largely unknown, and there is increasing interest in understanding their specific effects on the implicated cells/molecular factors. Particular attention is being focused also on their potential clinical application in chronic pathologies characterized by delayed and impaired healing, such as diabetes and vascular diseases in lower limbs. On these bases, we firstly summarized the current knowledge on wound healing (WH) in skin, both in normal conditions and in the setting of these two pathologies, with particular attention to the cellular and molecular mechanisms involved. Then, we critically reviewed the outcomes of recent research papers investigating the activity exerted by Omega-3 PUFAs and their bioactive metabolites in the regulation of WH in patients with diabetes or venous insufficiency and showing chronic recalcitrant ulcers. We especially focused on recent studies investigating the mechanisms through which these compounds may act. Considerations on the optimal dietary doses are also reported, and, finally, possible future perspectives in this area are suggested.

## 1. Introduction

For about thirty years, our main research interest was focused on the mechanisms underlying the anti-inflammatory and antineoplastic effects of the bioactive long chain Omega-3 polyunsaturated fatty acids (LC Omega-3 PUFAs) of dietary origin [1,2,3,4]. Over this long period of time, these natural compounds have attracted considerable attention worldwide as affordable and not harmful remedies for the prevention and cure of a wide range of chronic disorders, especially the inflammation-based ones. In recent times, the possible role of LC Omega-3 PUFAs as regulators of the complex and inflammation-related process of wound healing (WH) has become a subject of great interest [5,6,7]. The most bioactive LC Omega-3 PUFAs, eicosapentaenoic acid (EPA, 20:5 ω-3) and docosahexaenoic acid (DHA, 20:5 ω-3), can be obtained either from their main dietary sources, i.e., fish and algae, or indirectly through the endogenous metabolic conversion of their precursor, the shorter chain Omega-3 PUFA α-linolenic acid (ALA, 18:5 ω-3), found at high levels in vegetables and nuts. However, it has been observed that not all individuals have identical efficiency in metabolically converting ALA into EPA and DHA, in relation to the specific isoform of the enzymes (desaturases) constitutively possessed [8]. Thus, genetic characteristics and epigenetic modulation of the expression of these enzymes may render different individuals high or low producers of LC Omega-3 PUFAs from dietary ALA. On the other hand, in most cases, ALA itself has shown fewer anti-inflammatory properties [9] than EPA and DHA, even though some studies have shown that ALA metabolites (i.e., phytoprostanes, 13-hydroxy octadecadienoic acid and 13-oxo-octadecadienoic acid) possess anti-inflammatory activities [10,11,12].

The LC Omega-3 PUFAs have the peculiar capacity to compete for incorporation at the tissue and cellular level with the other main class of dietary PUFAs, the Omega-6 PUFAs, and often exert opposite effects on physiologic and pathologic processes, including inflammation. Several decades have already passed since LC Omega-3 PUFAs and their bioactive metabolites (resolvins, maresins, protectins), were recognized as powerful negative modulators of acute inflammation and/or inducers of its resolution [13]. However, the precise role exerted by these dietary compounds during the healing process is still controversial. Currently, there is increasing interest in discovering the effect that they may exert on the cells implicated in this process, as well as on the cytokines and growth factors produced by them. In turn, these cell-produced factors may drive the recruitment, activation and functions of the cells involved in the healing process. This peculiar activity of the LC Omega-3 PUFAs is currently considered of great interest in relation to their potential clinical application in chronic pathologies characterized by dramatically impaired and delayed healing processes [14].

On these bases, we firstly summarized here the current knowledge on the WH process in normal and pathological conditions, with particular attention to the cellular and molecular mechanisms involved. Additionally, we have critically reviewed the outcomes of recent research papers (published in the last few years, 2015–2021) investigating the activity exerted by LC Omega-3 PUFAs and their bioactive products in the regulation of WH occurring in skin, focusing particularly on altered WH in patients affected by chronic conditions such as diabetes and vascular diseases in the lower limbs. Within the specific context of our review, we especially focused on those reports investigating the mechanisms through which these compounds and their bioactive products may act, with particular attention to the most recent developments in this area of research. We focused here on the effects of treatments with EPA and DHA, but not on the effects of ALA treatments, due to relatively scarce anti-inflammatory, pro-resolving and pro-repairing effects shown by this shorter-chain Omega-3 PUFA [9].

Finally, based on more recent discoveries that have shed light on possible new routes through which Omega-3 PUFAs may exert their beneficial effects in other pathologic contexts, we also proposed future directions in this area of research.

## 2. WH Process in the Skin: Cells and Molecules Involved

Skin represents a multifunctional organ with the main role of protecting our body from different chemical, physical and biological stressors that often cause tissue injury. Our skin activates a complex process to allow efficacious and rapid wound repair. The mechanisms underlying this process may be impaired because of systemic pathologic conditions, such as those associated with aging, diabetes or venous insufficiency. Patients affected by these chronic conditions are at higher risk of developing chronic wounds that are increasingly of high clinical relevance in the population [15].

WH is divided into four main phases: hemostasis, inflammation, proliferation, and remodeling [16,17]. In each of these phases, different cell types are involved, each with specific functions in the healing process, which will be briefly described in the following 2.1–2.4 sections. 

### 2.1. Platelets and Endothelial Cells

Platelets exert their peculiar function mainly during the hemostasis phase. They become activated by the exposure of extracellular matrix (ECM) components [18] and adhere to the damaged vessel walls. Thrombin formed by the coagulation cascade induces platelet shape modification, degranulation, and release of molecules (i.e., ADP, thromboxane A2, etc.) able to recruit other platelets to the site of damage [19]. The main function of the clot is to avoid hemorrhage, preventing invasion by pathogens [20]. Platelets also release growth factors, which stimulate the functions of the cells participating in the healing process [21], including endothelial cells, which, at this stage, start to repair the blood vessel [22]. 

### 2.2. Resident Immune Cells 

Following a skin wound, the innate immune response is activated by signals represented by molecules released by necrotic tissue cells (damage associated molecular patterns, DAMPs) or molecules expressed by pathogens (pathogen associated molecular patterns, PAMPs). DAMPs and PAMPs stimulate the resident innate immune cells, such as macrophages, Langerhans cells and mast cells, through specific pattern recognition receptors (PRR) to activate inflammation [23,24]. Innate immune cells release a series of different cytokines and chemokines, which recruit circulating leukocytes to the site of the damaged tissue [25]. They also produce pro-inflammatory molecules, which induce vasodilation and the expression of adhesion molecules on endothelial cells, such as selectins, which facilitate the adhesion and diapedesis of neutrophils and monocytes [26].

### 2.3. Neutrophils and Monocytes/Macrophages 

Neutrophils are the first type of cell reaching the damaged site. They are recruited by cytokines such as interleukin-1 (IL-1), tumor necrosis factor-α (TNF-α) and by bacterial endotoxins, such as lipopolysaccharide (LPS) [27]. Neutrophils are professional phagocytes, able to remove cell debris and kill microbial pathogens thanks to their ability to produce high amounts of reactive oxygen species (ROS), as well as a series of peptides with microbicidal activity, eicosanoids, and proteolytic enzymes [28]. They also entrap and kill pathogens by the neutrophil extracellular traps (NETs), a DNA network covered by antimicrobic peptides and cytotoxic histones [29]. In the absence of an infection, within a few days, neutrophils decrease in number at the site of inflammation [30]. 

Then, chemokines recruit circulating monocytes in the damaged tissue where they differentiate into macrophages, playing a central role in both the first induction phase of the WH-related inflammation, as well as in the later phases of inflammation resolution, tissue regeneration and remodeling. Besides removing cell debris and microbial pathogens similarly to neutrophils, macrophages possess other peculiar characteristics, and their morphology and functions change depending on the cytokines that they are exposed to [31]. Usually, macrophages are classified into two main categories: the classically activated macrophages (M1) and the alternatively activated macrophages (M2). However, that the M1 macrophage phenotype is the inducer of the inflammatory response, whereas the M2 phenotype is the main player in later phases, represents a quite simplistic view that has now been complicated by recent findings showing the high intrinsic plasticity of these cells and their capacity of assuming not just two, but several different phenotypes depending on the stimuli to which they are exposed [32,33,34]. However, for the sake of simplicity, we will refer here to the M1 macrophages when speaking of the pro-inflammatory macrophages, and to the M2 macrophages when considering those with anti-inflammatory and pro-resolving activities. M1 macrophages, which are activated by LPS and IFN-γ, release ROS and pro-inflammatory cytokines (i.e., IL-1, IL-6, and TNF-α). The later stages of inflammation are characterized by the transition of macrophages towards the M2 activation pathway. This can occur both through the recruitment of new circulating monocytes or through the phenotypic switch of macrophages already present at the damaged site by the action of environmental cytokines [35], by miRNA expression [36], growth factor stimulation [37], as well as by the modulation of anti- and pro-inflammatory receptors [37,38]. M2 macrophages produce a series of anti-inflammatory and pro-resolving cytokines, such as IL-4, IL-10, IL-13 [39,40], and arginase, an enzyme playing a key role in the wound repair process [41]. They also release growth factors such as FGF and VEGF to induce the proliferation of epithelial cells, fibroblasts, and endothelial cells [42]. 

### 2.4. Keratinocytes, Fibroblasts and Myofibroblasts

In the proliferation phase, keratinocytes, fibroblasts, and macrophages cooperate to induce wound closure, ECM deposition, and neo-angiogenesis. Keratinocyte activation also involves a partial epithelial-mesenchymal transition (EMT), which confers to these cells a higher migratory ability [43]. Keratinocyte migration in the wound occurs through interactions between integrin receptors and structural proteins of the provisional ECM [44]. Moreover, matrix metalloproteinases (MMPs), particularly MMP-1 and MMP-9, or plasmin, are essential for keratinocyte migration due to their ability to dissociate the integrin receptor or to degrade fibrin present in the provisional wound [43,45]. When keratinocytes at the opposite margins of the wound meet, their migration stops, a thin epithelial layer is formed, and keratinocytes adhere to the underlying ECM. Then, keratinocytes undergo terminal differentiation and stratification to regenerate epidermis [46].

Fibroblasts represent the main cell type responsible for the replacement of the provisional fibrin-rich ECM with granulation tissue. These cells respond to molecules produced by platelets, endothelial cells, and macrophages, which lead to differentiation of fibroblasts into myofibroblasts and wound contraction [47]. Fibroblasts are also responsible for both the degradation of the provisional matrix by producing MMPs, and for the formation of a granulation tissue rich in fibronectin, immature collagens, and proteoglycans [48]. 

Angiogenesis occurs at this stage to ensure an adequate nutrient and oxygen supply to the highly proliferating tissue [49]. The process is induced by hypoxia, which, in turn, increases the expression of the hypoxia-inducible factors (HIFs) and COX-2, leading to the increased expression of pro-angiogenic factors, such as VEGF [50]. In response to these factors, endothelial cells proliferate and migrate to the wound site, and create new vessels that give rise to a stable network [51]. Macrophages play a key role also in angiogenesis, producing different growth and chemotactic factors (i.e., VEGF, TGF-β, TNF-α, etc.). Moreover, macrophages secrete MMPs to degrade the fibrin network [52], participate in the neovessel remodeling [42,53], and inhibit angiogenesis in order to avoid excessive vascularization [54]. 

Tissue repair always also involves the regeneration of skin nerve fibers. It has been observed that, during the repair process, sprouting neurons and immune cells release neuropeptides such as substance P, a molecule able to influence cell proliferation and angiogenesis [55,56]. It is interesting to underline that substance P appears reduced in diabetic wounds, and that its topical application restores healing [57,58] as well as nerve regeneration [59].

ECM remodeling represents the last phase in tissue repair. It represents a process through which a mature scar, rich in type I collagen, is formed [47]. Fibroblasts in this phase replace the initial fibrin clot with hyaluronan, fibronectin, and proteoglycans and, later in the repair process, produce mature collagen fibrils [60]. Unwounded adult skin is made of 80% type I collagen and 10% type III collagen. Conversely, granulation tissue is formed by 30% type III collagen and 10% type I collagen [61]. During the healing process, type III collagen is gradually replaced by type I collagen, which increases the scar tensile strength [62]. However, the ECM integrity and architecture will not be the same as in the unwounded skin, and scar tissue confers only 80% of the initial tissue strength [61,63]. The sequential ECM modifications need a regulated balance between collagen synthesis and degradation, and this is accomplished through the temporal modulation of MMP expression [60]. Elastin, another essential component of the ECM, must replace the elastic fibers to restore skin elasticity. Elastin is synthesized from its precursor, tropoelastin, and in the early phases of tissue repair shows a provisional arrangement. Only after several months from injury, mature elastin fibers appear in the scar [64,65]. 

The increased TGF-β levels at the wound site and the mechanical tension stimulate the differentiation of myofibroblasts [66]. Myofibroblasts are characterized by high levels of α-smooth muscle actin (α-SMA), a protein allowing these cells to generate powerful contractile strength [60]. Myofibroblasts, which adhere one to another through desmosomes, are responsible for ECM stabilization also by binding to matrix fibrils through a process called contracture [67]. The repair process ends as soon as macrophages, endothelial cells, and fibroblasts undergo apoptosis or leave the damaged site, where a scar has now replaced the injured tissue [68].

## 3. Impaired Healing in Diabetes and Venous Stasis Ulcers

Healing impairment related to the chronic conditions of diabetes and vascular diseases in the lower limbs represents the most frequent cause of persistent ulcers, characterized by the tendency to not heal after 3 months or remaining unhealed after a year with conventional treatments [69,70]. Due to this refractory behavior, they may deeply deteriorate the patients’ quality of life. Moreover, in diabetic patients, the impairment of the WH process represents a serious complication, and a critical concern, since chronic ulcers arising in the lower extremities increase the risk of infection, may require long hospitalization, and ultimately even lead to amputation [71]. 

It has been reported that up to one third of diabetic patients may develop lower extremity ulcers [72], and, among them, more than 10% may experience amputation [71], thus substantially enhancing (by 2.5 times) their risk of death at 5 years [73]. However, most lower extremity ulcers appear to be caused by vascular insufficiency (with venous insufficiency causing 50–60% of the cases and arterial insufficiency 15–20% of them), while only 5% are referred to diabetes [74]. Moreover, these lesions due to venous insufficiency show a high rate of recurrence (50–70% at 6 months), which can lead even to infirmity [75,76]. It should be underlined that all of these chronic, nonhealing ulcers are particularly common in aging people. In particular, the prevalence of venous stasis ulcers, which is up to 2% in the general population, increases to 4–5% among individuals aged 65 and over [77,78]. 

Over the last two decades, a wide body of literature has explored the mechanisms underlying the impaired WH occurring in both of these pathologic conditions. This has led to the identification of alterations in cells, cellular functions, molecular pathways, and molecules involved, all representing potential targets of innovative therapeutic strategies [79,80]. In particular, the prevailing current approach in studying the mechanisms underlying the physiological and pathological WH processes is that of focusing on the types/subtypes and functions of cells recruited or participating in the development and termination of the processes [17], as well as their possible qualitative or quantitative modifications leading to WH impairment and delay. This approach has also allowed the discovery of a series of molecular factors implicated in the recruitment/activation of these cells, as well as the mechanisms through which these factors become altered in the wound area. 

In the following two sub-paragraphs, we will summarize the main mechanisms invoked so far to explain the impaired WH of patients affected by diabetes (Section 3.1) and vascular alterations of the lower limbs (Section 3.2). 

### 3.1. Mechanisms of Impaired WH in Diabetes

Macrophages and their possible functional alterations in impaired WH of diabetes have attracted considerable interest, due to the critical regulatory role exerted by these cells in the healing process [81]. Multiple findings obtained in different preclinical mouse models have demonstrated that both the increased number of monocyte/macrophages in the diabetic wound area [82,83] and the altered transition of these cells from the predominant M1 to the M2 phenotype [83,84] contribute to the impaired WH occurring in diabetes [84]. For instance, in diabetic mice it was reported that there was an abnormal extension of the WH inflammatory phase following the 3rd–4th day post-injury, when the reparative phase is normally occurring [83,85,86]. This unusual extension has been linked to an abnormal second influx of M1 macrophages in the injured tissue, which leads to perpetuation of inflammation [86]. This means that, if, on one hand, the metabolic conditions related to diabetes induce the sustained arrival and accumulation of monocytes/macrophages from bone marrow, they also inhibit the M1 macrophage reprogramming to the reparative M2 phenotype that usually occurs in the wound area [84]. Two mechanisms have been recently invoked to explain the dysregulated number and phenotypes of macrophages, as well as of the other cells participating in the healing process. Both mechanisms are related to the microenvironmental changes observed during impaired WH. The first one focuses on the altered epigenetic regulation of the number and functions of macrophages, as well as the other cells actively participating in the WH process, which are believed to be induced by the excessive load of glucose in diabetes [85]. The second mechanism is related to local alteration of microbiota in the skin healing area in diabetes.

An exhaustive review was recently published on the topic of the epigenetic regulation of macrophage function during normal and impaired WH [87]. The increasing interest in this issue is demonstrated by the large number of recently published papers analyzed in that review. In particular, a large body of recently produced literature supports the hypothesis that a dysregulation of enzymes named “epigenetic modifiers” and of their functions may be primary events related to the altered expression of a series of genes involved in impaired WH. This in turn may be responsible for the alterations in the number and activities of macrophages and other cells participating in WH [88,89]. In this context, it is worth noticing the altered epigenetic regulation of bone marrow monocytic progenitor cells prior to their recruitment in the wound area, observed to occur in hyperglycemic conditions [85]. Among the epigenetic modifiers that have been observed to participate in the impaired regulation of WH, there are enzymes responsible for histone covalent modifications (acetylation and de-acetylation, methylation, etc.) [90,91] or DNA methylation/demethylation [92]. Similarly, many findings have supported the role that may be exerted in WH and its dysregulation by a class of modulators of gene expression acting at the post-transcriptional level, i.e., the non-coding micro-RNAs. More recent findings have also indicated the participation of a different class of non-coding RNAs in the pathogenesis of impaired WH, namely, the long non-coding RNAs, which are pleiotropic as they are able to modulate the activity of other non-coding RNAs (micro RNA and circular RNA), and to influence a wider series of cellular processes, such as chromatin organization, mRNA stability and transcription, as well as protein translational and post-translation modifications [93,94]. 

According to the second pathogenic mechanism, skin microbiota has been identified as a crucial microenvironment factor able to deeply influence the WH process both in negative and positive ways [17,95,96]. It is known that healthy human skin is colonized by a variety of commensal microorganisms that interact with other microbial species and cells located in the skin, including immune cells, thus protecting the skin from pathogens [97]. However, overgrowth of some of the commensal microbial species or infection by pathogens may produce a selective shift in colonizing bacteria, and a decreased microbial diversity that may affect the WH process in diabetes [98,99]. The altered microbiota may cause the sustained activation of the innate immune system [23] and the progression of the inflammatory process toward a chronic course [100], thus contributing to explain the conversion of the acute wound into a difficult-to-heal chronic ulcer. Moreover, as recently underlined in a review by Luqman and Götz [96], an injury in the skin induces the local and systemic production of adrenaline, which may delay the WH process through the binding and activation of β-receptors [96]. The sustained activation of specific subclasses of these receptors was reported to hamper the pro-healing activities of keratinocytes, immune cells, fibroblasts, and endothelial vascular cells [101,102,103,104]. Interestingly, it has been seen that the microbiota present in healthy balanced skin may inhibit a cascade of negative effects triggered by the binding of adrenaline to its receptors. For instance, a normal microbiota converts aromatic amino acids into trace amines, which may function as powerful antagonists of adrenalin β-receptors [96], and probably this protective effect may be lacking when the microbiota is subject to deep changes, such as in diabetic wounds. Conversely, microbiota may even exert a deleterious effect. For instance, it has been observed that nonhealing diabetic wounds are markedly colonized by bacteria, such as *Staphylococcus*, *Pseudomonas*, *Corynebacterium*, *Enterococcus*, or *Helcococcus.* Some of these species grow and produce biofilms able to increase microbe immune evasion and antibiotic resistance [105,106,107]. Interestingly, Kim et al. [99] related the high level of oxidative stress typical of diabetes to the decreased diversity of microbiota in chronic wounds. They suggested that the oxidative stress could be responsible for the prevalence of certain microbes, such as *Pseudomonas aeruginosa*, showing a high propensity to form thick biofilms that hamper WH. One alternative possibility is related to the fact that the spike in adrenaline after injury and the following activation of β-receptors may affect components of skin microbiota, increasing their ability to produce biofilms [108,109]. Moreover, products of skin bacteria prevailing in diabetes have been reported to play a relevant role in the pathogenesis of chronic ulcers. Among these, there is the PAMP muramyl dipeptide which, besides triggering inflammation through the activation of its ligand (the pattern recognition receptor NOD2 and of the NALP3 inflammasome), is a powerful inhibitor of epithelial regeneration [107,110]. It has been demonstrated [107] that the up-regulation of muramyl dipeptide and its anti-regenerative effect in skin could be mediated by the up-regulation of the antimicrobial peptides β-defensins in keratinocytes, particularly the β-defensin 14, which, besides affecting the local microbiota, may negatively influence the healing process.

### 3.2. Mechanisms of Impaired WH in Patients Affected by Chronic Venous Leg Ulcers

The non-healing venous ulcers arising in lower limbs are skin lesions arising especially in the ankle region. They are mainly related to raised pressure in deep and superficial veins of the legs [75]. The patients carrying these ulcers show venous insufficiency, which, in about two thirds of them, is of primary type and originating from varicose vein disease. Otherwise, the remaining portion of patients have secondary venous insufficiency arising from post-thrombotic syndrome [111]. Within the venous system, both macrovenous hemodynamic changes (venous valve dysfunction, reflux, or obstruction) leading to hypertension, and microvenous changes (endothelial cell dysfunction related to shear stress) have been implicated [80]. 

Since the prevalence rates of chronic leg ulcers are increasing, and conventional therapies are often ineffective [112,113], new therapeutic strategies are being investigated [76,80]. However, to conceive new approaches for therapy it is necessary to know exactly the mechanisms underlying the development and progression of these lesions. Mechanistic research focusing on WH in chronic venous leg ulcers, compared to that centered on WH in chronic diabetic ulcers, suffers from a lack of suitable animal models, and most of the studies are human trials or ex-vivo investigations conducted on molecular/cellular components obtained from patient wound areas and fluids. Nevertheless, a great advance in knowledge has been made in the last few decades. For this issue, readers can directly refer to a recently published comprehensive review [80] focused on the pathophysiology and treatment of chronic venous leg ulcers. However, for the sake of clarity, it is important to underline here that an altered regulation of a series of cells and molecular factors involved in the different phases of the healing process was recently implicated in the pathogenesis of chronic venous leg ulcers. In particular, leucocytes, platelets, endothelial cells, vascular smooth muscle cells, fibroblasts and keratinocytes have been shown to participate in the pathogenesis of venous chronic leg ulcer development [80]. 

According to the original trapping theory [114], a crucial pathogenic step resulting in a chronic nonhealing ulcer is the entrapment of leukocytes in the venous circulation affected by hypertension. Consequently, leukocytes are activated, and they adhere and transmigrate through the dysfunctional endothelium into the neighboring tissues, where they release pro-inflammatory factors and induce a sustained chronic inflammation accompanied by angiogenesis and remodeling. Interestingly, an abnormally increased number of activated neutrophils entrapped in the altered venous circulation and wound area had originally been thought to initiate the process involved in the development of chronic ulcers in these patients, by inducing sustained inflammation and delayed healing [115,116]. 

The molecular factors implicated in these processes have been found to be increased either in the healing area or in the circulation. Among them, there are endothelial adhesion molecules, nitric oxide (NO), ROS, MMPs, growth factors, and cytokines (such as TGF-beta, VEGF, PDGF, TNF-alpha), chemokines, and iron (for a review, see [80]). 

Moreover, plenty of data have demonstrated that nonhealing venous ulcers are characterized by a dysregulated proteolytic activity, mainly due to increased levels and activity of neutrophil elastases and MMP, and decreased levels and activities of tissue inhibitors of MMPs (TIMPs). A special role was attributed to the dysregulation of MMPs [80,117,118], which have the capability to act on extracellular proteins and, consequently, on the process of tissue remodeling. Their altered levels and regulation may lead to the disruption of the balance between ECM synthesis and degradation. Recently, it was demonstrated that the degree of expression of MMP-1 and MMP-2 evaluated in venous ulcer biopsies, as well as the decrease in their expression along the observation period (4 weeks) represented a predictor of a more successful healing of the venous ulcers evaluated [119]. Interestingly, the MMP dysregulation and the abundance of specific MMPs having a peculiar role in the pathogenesis of venous chronic ulcers (such as MMP-1 and MMP-8) have been especially associated [120] with the presence of microbial pathogens such as *Staphylococcus aureus, Corynebacterium striatum,* and *Pseudomonas aeruginosa* in the healing area*,* reinforcing the hypothesis that an altered microbiota also may be a precipitating factor in the chronicization of these lesions.

The metabolic approach used in investigating the pathogenesis of venous chronic ulcers and other venous disorders seems also particularly worth noting. It was reported in a systematic literature review recently performed by Onida et al. [121] that lactate BCAA, lysine 3-hydroxybutirate, and glutamate were the metabolites most often identified in biopsies taken from ulcers and venous ulcer fluids. This area of research seems very fruitful and promising for providing new insight into the pathogenic mechanisms of chronic venous ulcers. It also has the potential to allow a better understanding of how an altered metabolism may affect the participation of host cells implicated in the development of venous chronic ulcers, as well as the relationships between the host cells and a metabolic-sensitive microbiota. 

Finally, it is worth reporting here a new and stimulating pathogenic hypothesis that has been put forward recently by Bachar-Wikstrom et al. [74]. According to this hypothesis, increased endoplasmic reticulum (ER) stress in the cells participating in the healing process may exert a role in the development and progression of human chronic venous leg ulcers. The authors demonstrated that ER stress and the related upregulated unfolded protein response are peculiar features in biopsies obtained from patients affected by chronic venous leg ulcers. They observed biomarkers of an increased ER also upon examining the WH process developing in the elderly after an acute injury. This prompted them to suggest that the ER stress condition in the elderly may have a detrimental role and predispose to the development of chronic venous ulcers. They found a basal level of ER stress in keratinocytes and fibroblasts originated from biopsies isolated from normal aged and diseased leg donors as compared to the same cells from young donors, which instead did not show any basal ER stress. They also associated the increased ER stress to the observed decreased migration capacity. This observation is of value, since keratinocyte and dermal fibroblast migration plays a crucial role in the healing process and opens the possibility of using chemical chaperones, known to alleviate ER stress [74], to improve impaired healing of chronic venous ulcers.

## 4. Omega-3 PUFA Modulatory Effects on Impaired WH in Diabetes and Vascular Disease

As we have seen above, an extensive body of knowledge has been recently gathered on the WH process in healthy and pathological conditions, and in the last few years we have witnessed an upsurge of interest in identifying the possible mechanisms involved. Meanwhile, a considerable number of reports exploring the regulatory activities of Omega-3 PUFAs on the WH process in the skin have been published in an attempt to reveal the mechanisms underlying their beneficial action. 

On these bases, we have tried to focus here in particular on the mechanisms of WH that have been reported to be modulated by these fatty acids. Since different mechanisms have been implicated in the pathogenesis of impaired WH in patients affected by diabetes or chronic leg ulcers (see Section 4.1 and Section 4.2 above), we will critically analyze the reports focused on Omega-3 PUFA activity in these pathologic conditions in two different sub-paragraphs. 

### 4.1. Omega-3 PUFA Modulatory Effects on Delayed WH in Diabetic Patients 

In diabetic animals, superficial wounds experimentally obtained at skin level represent the main experimental model used to evaluate the ability of Omega-3 PUFAs to overcome the delay in the healing process typical of diabetes mellitus. 

In a recent report [122], Omega-3 PUFAs were administered intraperitoneally (i.p.) to diabetic rats by injecting 1 mL of Omegaven, containing 59.1 mg/mL EPA plus DHA. The Omega-3 PUFA treatment was performed both before and after the streptozotocin injection used to chemically induce diabetes (10 days before the start of the experiment and until its end). After providing excisional wounds at the level of the panniculus carnosus muscle of diabetic rats, the authors had to wait 7 days to observe significant morphologic differences between the Omega-3 PUFA treated or untreated rats at the level of wounds. Analyzing the wound areas in the Omega-3 PUFA treated rats, they found that the size of the area and the neovessels present in it were significantly reduced, indicating that a faster healing process was taking place. At the same time, they observed that the treatment with Omega-3 PUFA significantly increased the number of repairing fibroblasts present in the area and reduced the number of macrophages. The authors did not perform any phenotypic characterization of the residual macrophages at that time point. However, it is conceivable that, by then, the macrophage population present in the area had already switched toward a prevailing M2 phenotype, able to coordinate the late phases of the healing process. It is also presumable that the macrophage reduction in the WH area was related to the fact that they were already leaving the stage, in agreement with an Omega-3 PUFA-induced acceleration of the healing process. 

More recently, Jia et al. [123] treated for 2 weeks before wounding, and until the end of the experiment, streptozotocin-induced diabetic rats with a daily oral dose of DHA (100 mg/kg body weight). By using histological examination of samples taken from the wound area of diabetic rats, they found that the DHA treatment increased the number of M2 macrophages (CD206+) and decreased the number of pro-inflammatory M1 macrophages (iNOS+). They also evaluated the cytokines in plasma obtained from blood withdrawn at 6 days after injury and observed that the inflammatory index (the ratio between pro-inflammatory cytokine TNF-α and the anti-inflammatory cytokine IL-10) significantly decreased. Similar results were also obtained by evaluating the cytokines present in the supernatant of macrophages derived from bone marrow and activated for 2 days in vitro. 

Although we analyzed recent discoveries (published less than 5 years ago) related to research focused on Omega-3 PUFAs and WH, it should be underlined that pioneering works [124,125,126] had already shown the central role exerted by macrophages in the reparative phase of the healing process investigated in *db*/*db* diabetic mice treated with DHA metabolic derivatives (Resolvin D1 (RvD1); 14*S*,21*R*-dihydroxydocosa-4*Z*,7*Z*,10*Z*,12*E*,16*Z*,19*Z*-hexaenoic acid (14*S*,21*R*-diHDHA); protectin D1 (NPD1). However, at that time, the functional characterization of the different macrophage phenotypes was still in its infancy. 

It is interesting to emphasize that even before these findings were published and the role of Omega-3 PUFA as pro-healing factors could be definitively established, controversies had emerged on the possible null [127], positive [128,129], or negative [130] effect of Omega-3 PUFAs on WH. It should be noted, however, that where null or negative results were reported [130], the Omega-3 PUFA used was ALA, which is less bioactive than EPA or DHA, or WH was studied in normal cells [130] or in healthy patients treated with corticosteroids [127]. 

In the recent study by Jia et al. [123] analyzed above and reporting that Omega-3 PUFAs showed a high pro-healing efficacy, these fatty acids were orally administered, which is also the easiest way of supplementation for possible preventive or therapeutic use in humans. Hence, it is worth considering which dietary dose could potentially induce the same beneficial effects in humans. The authors [123] used the oral DHA of 100 mg/kg body weight, as it was previously shown that this dose induced considerable anti-inflammatory and antioxidant efficacy in rat models [131,132]. Interestingly, in an extensive and comprehensive review of the literature [133], we compared the doses of Omega-3 PUFAs given to animals and humans and underlined that, as observed by Bougnoux et al. [134], an oral dose of 2–3 g given to breast cancer patients was shown to be optimal for inducing antineoplastic effects in terms of clinical parameters such as increased overall survival, longer time to progression, and anti-proliferative effect. We observed that this human dose induced the same tissue enrichment observed in rats supplemented with 200–900 mg/kg, i.e., the range of doses able to optimally inhibit the growth of experimental tumors in rats. As we observed, this range of doses is from twice to about 9-fold higher than that used to overcome the alterations of an impaired WH process in diabetic rats according to the findings of Jia et al. [123]. This suggests that for efficient pro-healing activity in patients at risk or bearing recalcitrant ulcers, it could be sufficient to provide Omega-3 PUFA as a dietary supplement with a daily oral dose much lower than those (2–3 g of DHA or EPA+DHA/day) used in breast cancer patients to obtain antineoplastic efficiency. Actually, a recent human study [135] reported that a significant improvement in WH was obtained by treating diabetic patients carrying chronic foot ulcers with 2 g Omega-3 PUFAs per day over a period of 12 weeks. However, it is important to underline that, in this case, the patients were supplemented with flaxseed oil, which is particularly rich in ALA, the metabolic precursor of the more bioactive long-chain Omega-3 PUFAs, EPA and DHA, which is reported to be only partially converted to them inside the human body and showing lower anti-inflammatory activity [136]. This observation further supports our hypothesis that doses of highly bioactive Omega-3 PUFAs lower than those needed to potentially reduce tumor growth could be able to improve the efficiency of an impaired WH process in diabetic patients. These authors [135] found that, following the treatment, all ulcer size parameters (length, width, and depth) were markedly and significantly reduced. However, they did not evaluate the direct effect of the treatment on the local inflammation and repair process, i.e., they did not verify possible modifications that may have occurred either in the cellular components or molecular mediators implicated in the healing process. However, they observed some beneficial changes induced by the treatment on insulin metabolism (reduction of serum insulin and glycated HbA1c concentrations, increased insulin sensitivity), systemic inflammatory biomarker (reduction of hs-CRP levels), and oxidative status (increased plasma total antioxidant capacity, TAC, and GSH levels). Thus, they hypothesized that the Omega-3 PUFA treatment could have indirectly affected WH through an improved metabolic profile, but without discussing this relationship in deep detail. 

It is worth mentioning at this point a very interesting therapeutic strategy that has been developed in recent years, based on the anti-inflammatory activity of fish-derived Omega-3 PUFAs and consisting of treatment with intact fish skin grafts rich in long-chain Omega-3 PUFAs applied on wounds of diabetic patients to facilitate their healing. For instance, a recently performed small multicentric trial [137] applied fish skin grafts weekly for up to 12 weeks to refractory chronic foot ulcers of diabetic patients also treated with conventional therapy. They observed that the fish skin graft treatment significantly increased the percentage of completely healed wounds at 12 weeks (67% vs. 32% in control patients). Similar results were obtained by another small trial conducted by Woodrow et al. [138] in diabetic patients to evaluate the potential benefit derived from an intact fish skin graft rich in Omega-3 PUFAs applied following forefoot surgery in diabetic patients. Fish skin grafts were used for 6 weeks in the diabetic foot and an area reduction of more than 80% was found in wounds of less than 3 months duration. However, the decreased time to healing was less evident in older wounds (wound age over 3 months, 40% wound area reduction), suggesting that the treatment could be more efficacious if used earlier during the healing process. 

Finally, we expanded our research of the literature concerning altered WH in diabetes also to comprehend findings related to the effects of Omega-3 PUFAs in corneal keratopathy, since they furnish equally important information obtained at the epithelial level in diabetic patients. Corneal abnormalities, usually named corneal keratopathy, are quite frequent complications in diabetic patients, and can easily result in corneal ulceration. However, it should be underlined that together with reepithelization, nerve regeneration and neuropathy coexist in the corneas obtained from diabetic human cornea donors [139], implying that both keratopathy and nerve damage/repair play roles in keratopathy development.

The effect of a DHA-derived metabolite, the pro-resolving lipid mediator resolvin D1 (RvD1), has been recently studied in a model of corneal epithelial WH in diabetic mice [140]. Diabetes was induced in the animals by performing an intraperitoneal streptozotocin injection, and then the central corneal epithelium was removed. The authors found that topically applying RvD1 (100 ng/mL, 5 µL/eye) induced significant resolution of inflammation in the cornea and its regeneration. It was previously shown that a specific pro-resolving mechanism of RvD1 rescues macrophages from oxidative stress-induced apoptosis during efferocytosis through the repression of NADPH oxidase activation and the induction of antiapoptotic protein expression [141]. In agreement, the authors found that RvD1 also reduced, at the corneal level, the abnormal ROS formation typical of diabetes by inhibiting NADPH oxidase expression. This is interesting because ROS accumulation has been implicated in the development and progression of a series of complications in diabetes. Moreover, at the corneal level, RvD1 was found to reactivate Nrf2, a transcription factor altered by hyperglycemia. Nrf2 is normally induced by oxidative stress to counterbalance it, by activating the transcription of a series of antioxidant genes [142]. As a result, the synthesis of glutathione was increased at the corneal level, as well as the expression of antioxidant enzymes, such as MnSOD, NADPH-quinone oxidoreductase 1 (NQO-1), and heme oxygenase (HO-1). A final interesting finding was that RvD1 healing activity was highly specific, as it was abolished by an antagonist of its formyl peptide receptor 2 (FPR2). This observation is in agreement with multiple previous observations, demonstrating that RvD1 anti-inflammatory activity is specifically mediated by this receptor in the context of different inflammatory pathologies [143]. Accordingly, He et al. [144] had found that, in mice affected by streptozotocin-induced diabetes and treated to obtain an injury at the corneal level, the topical application of DHA combined with pigment epithelium-derived factor (PEDF) for 2 weeks markedly accelerated corneal epithelium WH (time to complete closure of epithelial injury, 49 days in DHA+PEDF rats vs. 61 days in control rats). They observed that treatment increased the number of macrophages, especially those of M2 phenotype, that, with their pro-repairing activity, could explain the beneficial effect observed. Moreover, the treatment also increased the density of corneal epithelial nerves, corneal sensitivity, and the volume of tears in diabetic eyes showing a high level of dryness. It is interesting that the combination of PEDF+DHA was used for its capacity to reduce the oxidative stress responsible for nerve degeneration in diabetes [145].

### 4.2. Omega-3 PUFA Modulatory Effects on Delayed WH in Patients Affected by Chronic Venous Leg Ulcers

The possible modulatory effects exerted by Omega-3 PUFAs on impaired WH in patients affected by chronic leg ulcers have been mainly investigated by the group of McDaniel et al. [76,146]. Several years ago, this group of authors [147] found that aged adults (average age about 64 years) affected by chronic venous leg ulcers showed a relatively high plasma Omega-6/Omega-3 PUFA ratio. This finding implied that the patients were consuming a diet rich in Omega-6 PUFAs, which are known to promote inflammation. On that basis, and because it was reported that increased intake of EPA and DHA may help in reducing high Omega-6/Omega3 ratios in humans and animals [148,149], the authors designed two consecutive double-blind controlled trials [76,146] where chronic venous leg ulcer patients were treated with an oral supplementation of EPA plus DHA. In the first one, the authors treated the patients with a daily dose of 3.0 g Omega-3 PUFA (2.5 g EPA and 0.5 g DHA), as they had previously shown that this dose significantly increased the level of these fatty acids in blood after a period of 4 weeks [150]. They found that the EPA plus DHA oral supplementation, given for 4 or 8 weeks as an adjuvant to conventional therapy, significantly increased plasma levels of both the fatty acids and decreased the level of the main Omega-6 PUFA, arachidonic acid (AA, 20:6 ω-3), always with more evident effects at 8 than at 4 weeks. Moreover, they observed that the treatment beneficially affected the ulcer microenvironment, predisposing ulcers to faster healing. In fact, the EPA plus DHA treatment reduced the number of activated neutrophils (expressing the CD66 marker) in the wound fluids and, correspondently, the effect was more visible at 8 than at 4 weeks. In agreement, the dietary treatment also reduced the production of MMP-8 and human neutrophil elastase (HNE), which are factors produced by these innate immune cells in the healing area. These inflammatory mediators are reported to increase in fluid and biopsies taken from chronic vessel ulcers [151], and to be present at significantly higher levels in chronic refractory wounds compared to acute wounds [152]. In this first small trial, the dose of 2 g/day EPA plus DHA was used, since in most cases this dose was previously observed to be needed to reduce inflammatory cell functions and inflammatory mediator production [153,154]. However, in clinical settings, each specific inflammation-based pathology may require a particular Omega-3 PUFA dosage for the improvement of biomarker levels and symptomatology, as shown by the reported higher dose of Omega-3 PUFA needed in the context of rheumatoid arthritis (3.5 g/day EPA plus DHA) [155]. Still, it should be underlined here that using relatively high doses of Omega-3 PUFAs is not always the better option. In fact, it has been suggested that Omega-3 PUFAs may have different effects, depending on the levels at which they are incorporated in tissues, with lower doses exerting antioxidant effects, and higher doses pro-oxidant effects [133]. 

The second trial is on-going and will be completed in 2024 (NCT03576989). In the report recently published by the authors to disclose the protocol of this trial [76], we noticed that the authors had decided to decrease the daily dose of EPA by 25% with respect to their first trial (1.87 g EPA instead of 2.5 g), and to double that of DHA (1.0 g DHA instead of the previously used daily dose of 0.5 g). However, the sum of the two fatty acids was almost unchanged (2.87 EPA plus DHA instead of 3 g, as in the first trial). In our opinion, the increase in the dose of DHA was very appropriate, since not only EPA, but also DHA may be endogenously converted to several metabolic derivatives showing powerful pro-resolving activities [156]. The aims of this second trial include the evaluation of metabolic bioactive derivatives of EPA and DHA, as well as cytokines, in untreated and treated patient blood and wound fluid. At the local level, the trial will also comprise the study of neutrophil activation in wound fluids, changes in size of the wound area, and the expression of molecular factors that may affect healing. Moreover, clinical parameters (recurrence, variables in blood, symptoms, and quality of life) will be evaluated. As a conclusion, the authors predict that treatment with EPA plus DHA in combination with conventional topical therapy could substantially improve the healing of chronic venous leg ulcers and open an innovative and safe therapeutic route to improve the quality of life of patients and reduce financial burdens. 

It is interesting to see how both these trials were devised to substantiate the authors’ hypothesis according to which neutrophil dysregulation is the main pathogenic component in the impaired healing of chronic venous leg ulcers. However, as we have seen in Section 4.2, a variety of mechanisms have so far been involved in the pathogenesis of venous leg ulcers [80]. Therefore, this could represent an exciting area of future research for scientists interested in Omega-3 PUFA beneficial effects or possible clinical applications in inflammation-based diseases, as well as for those searching for new therapeutic strategies to support or substitute for conventional yet unsatisfactory cures [80]. 

Accordingly, it was recently highlighted in the review by Shaydakov et al. [157] that in the research field of venous leg ulcers, there are still substantial gaps of knowledge that remain to be addressed. For instance, the authors underlined that for topical treatment of these ulcers, the published data appear insufficient, scattered, and marked by significant methodological weakness. The conclusions of the American Venous Forum Research Committee reported in that review bring attention to these gaps and encourage researchers to work harder to close them in the next future. In this respect, it is interesting to underline that increasing efforts have been made recently to encapsulate purified Omega-3 PUFAs in nanomaterials [4]. These innovative products are designed to protect Omega-3 PUFAs from oxidative deterioration, as well as improve their delivery to specific target tissues (either alone or in combination with other bioactive natural products or drugs). Of course, they may also help in developing formulations lacking unpleasant odors and suitable for topical applications. 

## 5. Conclusions and Future Perspectives 

We can conclude that, based on the recognized anti-inflammatory activities of dietary Omega-3 PUFAs and their metabolic derivatives, and the results obtained so far in research on diabetic and venous leg ulcers, these compounds are considered by many authors to be potentially promising candidates for the prevention and treatment of these chronic disorders. 

**Figure 1:**
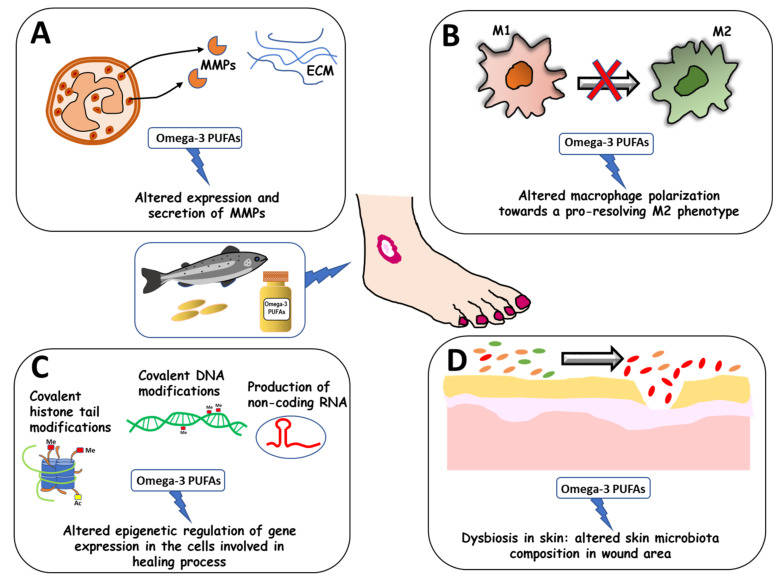
Future perspectives in research on nonhealing ulcers and Omega-3 PUFAs. Possible roles of Omega-3 PUFAs in the following altered processes that hamper and delay healing in diabetes or chronic venous leg ulcers: (**A**) altered expression/secretion of MMPs by innate immune cells; (**B**), altered polarization of macrophages; (**C**), altered epigenetic modulation of gene expression; (**D**), altered microbiota skin composition.

However, from a systematic point of view, it can be concluded that even though several mechanisms implicated in the pathogenesis of recalcitrant ulcers may be involved in the pro-healing activity of Omega-3 PUFAs, they remain poorly investigated. For instance, there is quite a large consensus that one crucial mechanism involved in the pathogenesis of recalcitrant venous ulcers is the activation of immune cell-derived proteases, and specifically neutrophil elastases and MMPs [152]. Interestingly, however, the available results on a possible MMP-suppressing activity of Omega-3 PUFAs remain scarce in this area of research [146] (Figure 1A). In our opinion, this issue deserves further attention in the future. In fact, results obtained in other pathological contexts have strongly supported the hypothesis that one route through which these fatty acids may act is the suppression of MMP production/secretion. For instance, it was observed that Omega-3 PUFA anti-migration and anti-invasive effects in cancer could be partly related to their ability to suppress the expression/secretion of these proteases by cancer cells [158,159,160,161]. Moreover, a relationship between the protective anti-aging effect of Omega-3 PUFAs on skin and their MMP activity-inhibiting effect also has been established [160,161,162]. 

Another possible mechanism that seems not to have been adequately explored regards the ability that these fatty acids may have to direct the polarization of macrophages toward a pro-resolving M2 phenotype in the diabetic wound area (Figure 1B). Findings obtained in other pathological settings have demonstrated the regulatory activity that these fatty acids may exert on macrophage polarization, thus helping to explain their beneficial effects [2]. For instance, it has been recently demonstrated that the benefits observed following treatment with Omega-3 PUFAs (or their metabolic derivatives) in cardiovascular diseases [163,164], acute lung injury [165], neurodegenerative diseases [166,167], and other inflammation-based diseases [2] can be partially related to their M2 macrophage-promoting polarization. Moreover, it appears that there is still plenty of room for further research in this direction because, according to recent findings, macrophages possess very high intrinsic plasticity and may assume additional phenotypes other than the M1 and M2 variants [168].

A further stimulating area of research in the field of impaired chronic WH regards the altered epigenetic regulation of gene expression in the cells involved in this pathologic process. The increasing knowledge obtained in the field of epigenetics in the last few years has allowed the identification of new mechanisms underlying the altered functions of the cells involved in nonhealing skin ulcers. Future work in this field will be necessary to establish which alterations involving epigenetic modifiers, or whether the epigenome itself, may be mainly responsible for the abnormal functions of these cells. This is extremely important because the epigenetic modifiers could have the potential to become new and useful prognostic biomarkers and/or therapeutic targets. In this context, it appears that the effects of Omega-3 PUFAs also should be investigated, since they already have been identified as mediators of epigenome reprogramming in other settings [2,169,170] (Figure 1C).

Finally, a large body of literature shows that the protective effects exerted by Omega-3 PUFAs in various diseases may be in part related to their capacity to affect gut microbiota composition and dysbiosis [171]. These fatty acids are reported to influence environmental factors (such as antibiotic, drug, or overnutrition stress) that are able to upset the balance of gut microbiota, causing dysbiosis [8,171,172,173,174,175,176] and, in turn, induce the development of a series of pathologies. As we noted (Section 3.1), several studies have demonstrated decreased diversity and dysbiosis of the microbiota present at the level of the injured skin. These studies have associated an unbalanced microbiota with the development of recalcitrant ulcers. On these bases, it might be worth investigating if an Omega-3 PUFA treatment may also positively affect skin microbiota dysbiosis in chronic ulcers, and through this mechanism, protect patients from the development of these recalcitrant disorders (Figure 1D).
